# 一种快速检测UGT1A1^*^28基因多态性的方法

**DOI:** 10.3779/j.issn.1009-3419.2017.12.04

**Published:** 2017-12-20

**Authors:** 迎 黄, 健 苏, 小穗 黄, 丹霞 卢, 至 谢, 素清 杨, 伟浜 郭, 志异 吕, 红穗 吴, 绪超 张

**Affiliations:** 510080 广州，广东省人民医院，广东省医学科学院医学研究部，广东省人民医院肺癌研究所转化医学重点实验室 Guangdong General Hospital Institute of Lung Cancer, Key Laboratory of Translational Medicine, Medical Research Center, Guangdong General Hospital Guangdong Academy of Medical Sciences, Guangzhou 510080, China

**Keywords:** UGT1A1, 片段分析, 基因多态性, UGT1A1, Fragment analysis, Polymorphism

## Abstract

**背景与目的:**

尿苷二磷酸葡萄糖醛酸转移酶（uridine diphosphate glucuronosyl transferase 1A1, UGT1A1）是伊立替康代谢最主要的同工酶，*UGT1A1*基因的多态性影响UGT1A1的活性，本研究建立片段分析法检测UGT1A1^*^28 TATA盒的多态性。

**方法:**

调取2014年4月-2015年5月在广东省人民医院的住院肺癌患者库存血液标本286例，建立片段分析法检测UGT1A1^*^28 TATA多态性，与Sanger测序方法比较其精确度和准确度。

**结果:**

286例肿瘤血液中，UGT1A1^*^28 TATA盒TA6/6型有236例（82.5%），TA6/7型有48例（16.8%），TA7/7型2例（0.7%）。片段分析法与Sanger测序法比较，精确度与准确度达100%。

**结论:**

片段分析法适用于临床检测UGT1A1^*^28多态性，成本低且方便快捷。

## Abstract (en)

**Background and objective:**

Uridine-diphosphoglucuronosyl transferase 1A1 (UGT1A1), UGT1A1^*^28 polymorphism can reduce UGT1A1 enzymatic activity, which may lead to severe toxicities in patients who receive irinotecan. This study tries to build a fragment analysis method to detect UGT1A1^*^28 polymorphism.

**Methods:**

A total of 286 blood specimens from the lung cancer patients who were hospitalized in Guangdong General Hospital between April 2014 to May 2015 were detected UGT1A1^*^28 polymorphism by fragment analysis method.

**Results:**

Comparing with Sanger sequencing, precision and accuracy of the fragment analysis method were 100%. Of the 286 patients, 236 (82.5% harbored TA6/6 genotype, 48 (16.8%) TA 6/7 genotype and 2 (0.7%) TA7/7 genotype.

**Conclusion:**

Our data suggest hat the fragment analysis method is robust for detecting UGT1A1^*^28 polymorphism in clinical practice. It's simple, time-saving, and easy-to-carry.

尿苷二磷酸葡萄糖醛酸转移酶1A1（uridine diphosphate glucuronosyl transferase 1A1, UGT1A1）是伊立替康（irinotecan）的主要代谢酶，其活性与伊立替康的疗效及不良反应密切相关，而UGT1A1^*^28基因多态性可显著影响该酶的表达与活性，增加伊立替康化疗患者发生腹泻、严重粒细胞减少等不良反应的风险。伊立替康是小细胞肺癌标准二线治疗方案，检测UGT1A1^*^28基因多态性对于指导肺癌个体化治疗具有一定临床意义^[1-4]^。

目前检测基因多态性常用的方法主要包括：Sanger测序法、变性高效液相色谱（denaturing high performance liquid chromatography, DHPLC）、变性梯度凝胶电泳（denatured gradient gel electrophoresis, DGGE）、单链构象多态性（single-strand conformation polymorphism, SSCP）、荧光定量PCR、PCR-LDR等多种。Sanger测序法是检测UGT1A1^*^28突变的传统和经典方法，但Sanger测序法耗时，且灵敏度低，仅达10%^[5, 6]^。本研究尝试用片段分析方法，以期快速、简便地检测UGT1A1^*^28多态性，获得一种更适用于临床的检测方法。

## 1 材料与方法

### 1.1 主要试剂和仪器

外周血DNA提取试剂盒（Qiagen公司），缓冲液、超纯甲酰胺、POP4胶和GeneScan-500 LIZ Size Standard（美国Applied Biosystems，ABI公司）。3730基因分析仪（美国Applied Biosystems，ABI公司），QIAsymphony SP高通量全自动样品纯化工作站（Qiagen公司），Nano Drop 8000分光光度计（Thermo公司），PCR仪（北京东胜创新生物科技公司），水平电泳仪（德国BIO-RAD公司），UVI凝胶成像系统（德国BioRad公司）。

### 1.2 样本及质控品

286例样本均取自广东省人民医院2014年4月-2015年5月住院肺癌患者，抽取静脉EDTA抗凝全血1 mL。患者临床特征见表 1，所有患者均签署知情同意书；10份UGT1A1^*^28质控品来源于国家卫计委。

**1:** 286例患者的临床特征及UGT1A1^*^28多态性 Characteristics of patients with lung cancer and UGT1A1^*^28 polymorphism

Characteristics	Cases	%
Age, median (yr, range)	51 (29-84)	
Gender		
Male	197	68.9
Female	89	31.1
Smoking status		
Non-smoking	139	48.6
Smoking	147	51.4
History		
AC	181	63.3
SCC	67	23.4
SCLC	17	5.9
Others	21	7.3
Stage		
Ⅰ-Ⅲ	147	51.4
Ⅳ	138	48.3
UGT1A1^*^28		
TA6/6	236	82.5
TA6/7	48	16.8
TA7/7	2	0.7
AC: adenocarcinoma; SCC: squamous cell carcinom; SCLC: small cell lung cancer.		

#### 1.2.1 引物合成

片段分析引物：上游引物：5’FAM-CTCCCTGCTACCTTTGTGGACTGA3’，下游引物：5’TAGCACCTGACGCCTCGTTGT3'；扩增产物为128 bp。Sanger测序上游引物：5’CTCCCTGCTACCTTTGTGGACTGA3’；下游引物：5’ACAACGAGGCGTCAGGTGCTA3’均由Life公司合成。

#### 1.2.2 DNA提取

按试剂说明书提取基因组DNA，测OD值，并配成10 ng/µL。

#### 1.2.3 PCR扩增

总反应体系10 µL，含Premix Ex Taq Hot Start Version 5 µL（大连TAKARA公司），DNA模板1 µL，2 µM上下游引物各0.5 µL，ddHO_2_ 3 µL。扩增条件为：94 ℃预变性3 min，94 ℃变性30 s，55 ℃退火30 s，72 ℃延伸1 min，共30个循环。反应结束后4 ℃保存。每次实验均加一个无模板空白对照。

### 1.3 基本扫描分析

取荧光标记的PCR产物1 µL，加69 µL水作1:70稀释，取1 µL稀释产物与0.5 µL Genescan-500 LIZ Size Standard及9 µL去离子甲酰胺混合，在ABI-3730 DNA序列分析仪中进行毛细管电泳。计算机自动收集电泳过程中不同时间所出现的不同颜色和强度的荧光素，以显示出不同的位置、高度、颜色和形态的峰；Gene Scan Genotyper 3.5软件自动分析收获的数据。

### 1.4 确定方法学的准确度、精确度及最低检测限

将DNA配制成不同的浓度（1 ng/µL、2.5 ng/µL、5 ng/µL、10 ng/µL、20 ng/µL），每个浓度检测三个复孔，以确定检测方法的最低检测限。所有样本及质控品均用Sanger测序法同时检测以确定检测的准确度；取4份样本由两个操作者分两个批次进行检测以确定重复性。

### 1.5 Sanger测序

使用本室建立起来的方法，简述如下：PCR反应体系：每个25 µL的PCR反应包括1 µL（20 ng/µL）模板DNA，12.5 µL Premix Ex Taq HS酶（大连TAKARA公司），1 µL正向和反向引物混合物，10.5 µL ddH_2_O。PCR反应条件为：初始变性94 ℃ 5 min：变性94 ℃ 30 s，退火55 ℃ 30 s，72 ℃延伸1 min，35个循环；72 ℃延伸7 min。将扩增的PCR产物经2%琼脂糖凝胶电泳检测目的片段符合条件后，纯化产物。纯化的PCR产物用BigDye Teminator V3.1按操作说明书进行标记及纯化，然后在ABI-3730测序仪上进行双向测序，测序结果用Chromas 2.31软件分析，人工校读分析UGT1A1上游启动子序列A（TA）nTAA中的TA重复序列。

### 1.6 样本检测

先取20例样本作为实验集，同时用片段分析法及Sanger测序法检测UGT1A1^*^28多态性，确定三种基因片段在毛细管电泳时的位置；其余266例为验证集，与10份质控品一起同时用片段分析法及Sanger测序法检测UGT1A1^*^28多态性。

## 2 结果

### 2.1 临床特征

286例肺癌患者的临床特征见表 1，肺腺癌181例，鳞癌67例，小细胞肺癌17例，其他病理类型21例。17例小细胞肺癌中，7例放弃治疗；在10例接受了化疗的患者中，有3例分别接受一线、二线和三线依立替康治疗，均无不可耐受的严重不良反应，基因型均为TA6/6型。

### 2.2 片段分析数据判读

电泳结束后，GenScan3.5软件自动分析生成的图谱文件，给出各片段位置、峰值大小。由于PCR引物标记上了VIC荧光，UGT1A1不同的分子分型在毛细管电泳图上产生位置和峰高不同的绿色产物峰。通过实验集的20例样本确定*UGT1A1*三种多态性的峰图特征：单个124.2大小的峰为TA6/6型，单个126.4大小的峰为TA7/7型，124.2及126.4两个峰为TA6/7型（图 1）。

**1:**
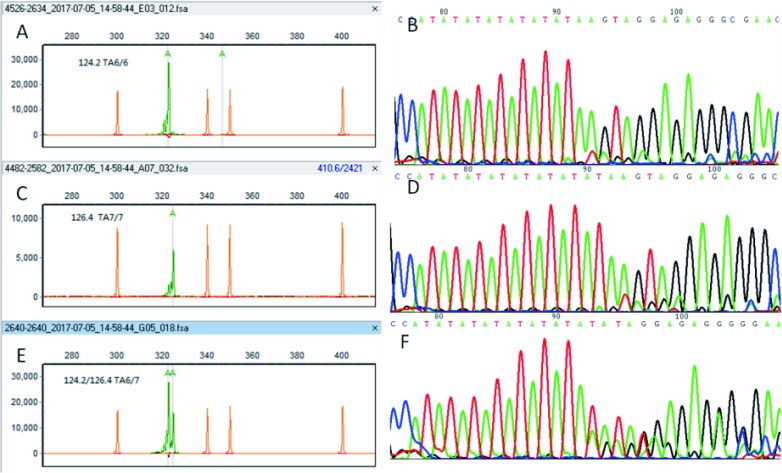
UGT1A1^*^28基因分型图例。A、B：UGT1A1^*^28 TA6/6野生型，峰的位置位于124.2；C、D：UGT1A1^*^28 TA 7/7纯合突变型，峰的位置位于126.4；E、F：UGT1A1^*^28 TA6/7杂合型，有两个峰，位置分别为124.2及126.4；A、C和E是片段分析图谱；B、D及F是Sanger测序图谱。 Representative figures of UGT1A1^*^28 polymorphysin. A, B: UGT1A1^*^28 TA6/6. The trace is located in 124.1; C, D: UGT1A1^*^28 TA 7/7. The trace is located in 126.4; E, F: UGT1A1^*^28 TA6/7. There are two traces which are located in 124.2 and 126.4, respectively. A, C and E are generated with fragment analysis, while B, D and F are generated with Sanger sequencing.

### 2.3 片段分析

片段分析10个质控品结果完全正确（表 2）。DNA最少上样量为1 ng/µL，不同操作者两批次检测的结果完全一致。

**2:** 片段分析检测10例UGT1A1质控品结果 Results of 10 standards detected with Sanger sequencing and Fragment analysis

ID	Sanger sequencing	Fragment analysis
1, 611	TA6/6	TA6/6
1, 612	TA6/6	TA6/6
1, 613	TA6/6	TA6/6
1, 614	TA7/7	TA7/7
1, 615	TA6/6	TA6/6
1, 621	TA6/7	TA6/7
1, 622	TA6/7	TA6/7
1, 623	TA6/6	TA6/6
1, 624	TA6/6	TA6/6
1, 625	TA6/6	TA6/6

### 2.4 两种方法一致性结果

286例样本通过片段分析法检测出UGT1A1^*^28为TA6/TA6 236例（82.5%），TA6/TA7为48例（16.8%），TA7/7为2例（0.7%）；与Sanger测序结果呈一致性，准确度达100%。10份质控品结果百分之百正确。

## 3 讨论

本研究建立一种片段分析法快速检测UGT1A1^*^28多态性，一共检测286例肺癌患者血液标本，与Sanger测序比较，准确性均达100%，参加国家卫计委室间质评结果完全正确。最少DNA用量可达1 ng/µL。3例小细胞肺癌患者分别接受一线、二线和三线依立替康治疗，均无不可耐受的严重不良反应，基因型均为TA6/6型。

UGT1A1启动子区域的TATAA区若插入一个TA，即A（TA）7TAA；野生型则为A（TA）6TAA。UGT1A1^*^28多态性有三个基因型：TA6/TA6（野生型）、TA6/TA7（杂合突变型）和TA7/TA7（纯合突变型）。TA6/7和TA7/7基因型的UGT1A蛋白质的代谢中间产物SN-38的糖脂化作用下降，因而增加了伊立替康的毒副作用，临床通过减少药物用量，可以减轻毒副作用，但对药物疗效影响不大，因此，UGT1A1^*^28基因的多态性有助于指导临床用药^[1-4]^。

目前，检测UGT1A1^*^28基因的多态性的方法主要是Sanger测序法^[7, 8]^，但Sanger测序法步骤繁琐、一个检测周期需要两天，结果判读较复杂，不能为临床提供快速的检测服务。

片段分析方法基本工作原理是用荧光标记的引物扩增DNA目的片段，若目的片段由于突变导致片段大小发生改变，将荧光标记的PCR产物置于ABI-3730基因分析仪上进行毛细管电泳，电泳结果用GenScan 3.5软件进行分析，电泳图上出现两个位置不一样的峰形，根据产物峰的位置大小可作出结果判断。毛细管电泳分辩率高，可以区分1个碱基的差异。由于此技术操作简便、耗时短、费用少，已得到广泛的应用。Furtado等^[9]^在真性红细胞增多症样本中比较Sanger测序法、熔解曲线法（high resolution melting, HRM）及片段分析法检测*JAK2*基因12外显子的突变，包括缺失突变、插入突变、K539L点突变，发现片段分析更灵敏，可检测突变含量为2%的样本（极限为0.5%），而HRM法及Sanger测序灵敏度则为10%；片段分析法结果解读更容易。该研究者设计了单管检测JAK2基因缺失突变、插入突变、K539L突变，提高了检测通量，缺点是不能同时检测其他类型的点突变。Gardner等^[10]^在47例血液肿瘤标本中比较了片段分析法、Sanger测序法及二代测序法检测*CALR*基因外显子9的缺失及插入突变。发现1例缺失突变，2例插入突变；片段分析法与Sanger测序法结果完全一致，二代测序法用常规流程检测时，该1例缺失突变未能检测，用IGV软件分析，发现是因为该区域的复盖度不够导致结果为阴性。但IGV可同时发现1个小缺失及几个点突变。

UGT1A1^*^28基因在启动子区域的TATAA盒在野生型为6个TA，其多态性插入了一个TA，野生型及突变型DNA片段之间相差两个碱基，在毛细管电泳时可以明显区分开来。本研究中所建立的片段分析法DNA用量少，可节约样本量；检测通量高（一次可检测96个样本），检测周期短，一个检测周期仅需1天；精确度及准确度均达到100%；成本低，适用于临床检测。近几年，虽然二代测序法以更高的灵敏度及更高的通量广受瞩目，但也因其存在一定的错误率及高费用、高技术要求，让基层单位望而却步。

毛细管电泳时，DNA产物的上样量对电泳图谱的质量影响较大，上样量过少，产物峰过低，可能会导致实验失败；若上样量过大，背景过高可导致误判结果。用水稀释PCR产物至一合适浓度，使产物的峰高与分子量标记LIZ500的峰高相当，则电泳图谱为最佳。

有研究^[11, 12]^认为：依立替康毒副作用与UGT1A1^*^6多态性有强相关关系而与UGT1A1^*^28多态性非相关；也有研究指出在亚裔患者中，依立替康毒副作用与UGT1A1^*^6/*28*均非相关。由于本研究小细胞肺癌的病例数较少，且没有检测UGT1A1^*^6多态性，未能阐明中国小细胞肺癌患者依立替康毒副作用与UGT1A1^*^6/*28*多态性的相关性。

综上所述，片段分析法适用于临床检测UGT1A1^*^28多态性，成本低且方便快捷。
